# MinION Adapted tNGS Panel for Carnivore Pathogens Including SARS-CoV-2

**DOI:** 10.3390/pathogens15010023

**Published:** 2025-12-24

**Authors:** Nelly O. Elshafie, Jobin J. Kattoor, Janetta Kelly, Rebecca P. Wilkes

**Affiliations:** 1 Willie M. Reed Animal Disease Diagnostic Laboratory, Department of Comparative Pathobiology, College of Veterinary Medicine, Purdue University, West Lafayette, IN 47907, USA; nelshafi@purdue.edu; 2 Department of Infectious Diseases and Microbiology, School of Public Health, University of Pittsburgh, Pittsburgh, PA 15213, USA; jok314@pitt.edu; 3 Indiana Department of Natural Resources, Division of Fish and Wildlife, Bloomington, IN 47401, USA; jkelly1@dnr.in.gov; 4 Veterinary Diagnostic Laboratory, University of Kentucky, Lexington, KY 40511, USA

**Keywords:** diagnostics, targeted next-generation sequencing, MinION, Oxford Nanopore Technologies, ONT, SARS-CoV-2

## Abstract

Affordable, flexible surveillance tools are needed to detect SARS-CoV-2 and other pathogens in wildlife. Standard nucleic acid amplification tests (NAATs) are reliable but restricted to predefined targets, limiting their ability to detect co-infections or emerging pathogens. To address this, we adapted a targeted next-generation sequencing (tNGS) panel for mesocarnivores to the Oxford Nanopore Technologies (ONT) MinION platform and combined it with a SARS-CoV-2 whole-genome sequencing assay. Merging both assays before library preparation enables simultaneous SARS-CoV-2 detection, variant identification, and broader pathogen screening. The MinION platform also improves turnaround time because sequencing can begin immediately on small numbers of samples, reducing costs in low-volume workflows. We converted our validated carnivore tNGS panel from the Ion Torrent system to MinION, optimizing amplification conditions, primer pools, and barcoding for multiplexing. Analytical sensitivity was measured using contrived wildlife samples spiked with serial dilutions of SARS-CoV-2 and tested in parallel with a commercial NAAT. Diagnostic sensitivity was assessed using contrived positives, and specificity was evaluated using NAAT-negative wildlife samples and in silico analyses. All 161 wildlife samples were NAAT-negative. MinION tNGS detected SARS-CoV-2 down to Ct 34 and produced ≥ 99% genome coverage for Ct ≤ 24 while simultaneously identifying additional pathogens. Diagnostic sensitivity and specificity were 96.7% and 100%. This workflow offers a low-cost, scalable approach for comprehensive wildlife pathogen surveillance.

## 1. Introduction

The development of robust diagnostic tests is essential for the surveillance and characterization of emerging diseases [1,2,3], particularly in cases where viral evolution may occur due to spill over into new host species [2,4]. This need is especially critical in wildlife species, which have the potential to serve as reservoirs for zoonotic viruses, but remain largely understudied [5]. The limitations of existing wildlife surveillance strategies have been highlighted by past outbreaks, such as the emergence of West Nile Virus (WNV) in North America in 1999 [6]. The delayed recognition and response to WNV underscored critical shortcomings in early detection efforts, as the virus had already established itself in avian and mosquito populations before becoming a significant public health threat.

Despite the implementation of targeted surveillance programs, such as the WNV dead bird surveillance program, these efforts primarily focused on detecting a single pathogen using rapid diagnostic tools, including PCR and immunohistochemistry. While effective for monitoring WNV transmission, this pathogen-specific diagnostic approach overlooked the potential presence of other avian diseases and arboviruses within the collected samples [7], representing a missed opportunity to broaden surveillance insights and improve preparedness for future outbreaks.

A similar challenge exists in detecting SARS-CoV-2, where nucleic acid amplification tests (NAATs) remain pathogen-specific despite being the gold standard for viral detection and may not account for infections with other clinically and epidemiologically significant viruses, such as the highly pathogenic avian influenza virus [8]. Expanding diagnostic capabilities to detect and differentiate multiple pathogens within a single assay would significantly enhance disease monitoring from a One Health perspective. Furthermore, integrating sequencing into the detection process allows for simultaneous virus detection and genomic characterization, reducing the turnaround time required to respond to emerging threats.

Real-time genomic sequencing provides vital information about infectious diseases. It helps identify when and where a virus was introduced. The data can show how the virus spreads across regions and what factors influence that spread. Researchers can also measure the genetic diversity of the virus. This allows for the detection of specific strains or genotypes that may cause larger outbreaks. Additionally, genomic data can support vaccine strategy planning by highlighting areas with high transmission. Overall, this technology is key to building effective surveillance systems that track disease movement across locations [9,10]. Targeted next-generation sequencing (tNGS) presents a promising approach to addressing these challenges; however, its high cost has historically limited its widespread use, particularly in wildlife surveillance.

The Oxford Nanopore Technologies (ONT)/MinION is one of the most promising sequencing platforms for wildlife surveillance testing due to its low cost and portability, which also allows its use in both the diagnostic laboratory and in the field [11]. Sequencing a sample in real-time is an added benefit, and with the use of a flongle flow cell, the cost is not prohibitive. The MinION is a third-generation sequencing technology that employs a nanopore-based technique capable of generating long reads of tens to hundreds of kilobases [12], and is touted for this ability when sequencing difficult areas of the genome, such as tandem repeats [13] or entire viral genomes [14,15,16,17]. Additionally, the platform can also be used with a targeted method to amplify and sequence much smaller pieces of DNA [13]. Enrichment of pathogen DNA using PCR targeting can increase the sequencing depth, thus influencing the attainable consensus accuracy [18], as well as increase the sensitivity of detection [19].

In our study, we utilized SARS-CoV-2 for proof of concept to develop a cost-effective tNGS protocol capable of detecting and characterizing SARS-CoV-2 while also enabling the identification of other relevant pathogens, as it would benefit both animals and humans. This approach aims to close critical gaps in pathogen surveillance by providing a more comprehensive, adaptable, and scalable method. We propose converting our carnivore pathogen tNGS panel [20] (including SARS-CoV-2) from a more costly short-read platform to ONT. Additionally, we propose adding the ability to sequence SARS-CoV-2 concurrently in the same test to reduce turnaround time and act on any important discovery. This study meets the need to develop a test for detecting and characterizing SARS-CoV-2 and other pathogens that may be significant to animal and human health, using a cost-effective protocol that can be shared across laboratories, including those with limited resources.

## 2. Materials and Methods

### 2.1. Animal Samples

One hundred sixty-one prospectively collected mesocarnivore samples (bobcats, badgers, raccoons, opossums, river otters, and skunks), including carcass tissues (lung, trachea, lymph node, spleen, small intestine, colon), oropharyngeal/rectal swabs, and feces were sourced from the Indiana Department of Natural Resources Indianapolis, IN, or a wildlife rehabilitation center in Bellingham, WA, and submitted to the Willie M. Reed Animal Disease Diagnostic Laboratory, Purdue University, West Lafayette, IN, USA. All samples were tested with an FDA EUA–authorized RT-PCR for SARS-CoV-2 following manufacturer’s instructions [TaqPath™ COVID-19 Combo Kit (ThermoFisher Scientific, Waltham, MA, USA)], which targets the ORF1ab, N, and S genes. A set of SARS-CoV-2 nucleic acids representing Omicron subvariants was obtained from human samples and provided by the Indiana Public Health Laboratory (Table 1). The nucleic acids were stored at −80 °C until testing. These nucleic acids were used to spike RT-PCR negative wildlife samples. Of the 161 samples obtained for this study, six samples (three bobcats, two raccoons, and a skunk) were used for evaluating different extraction kits. These samples were also spiked with variable amounts of the SARS-CoV-2 nucleic acids for evaluation of RT and Polymerase kits, for inclusivity testing, and for a SARS-CoV-2 targeted whole genome sequencing limit of detection study. A set of 30 of the samples was used for the diagnostic sensitivity testing, and a separate set of 30 samples was used for the diagnostic specificity study. The remaining 95 were used for a wildlife surveillance pilot using the validated method on the ONT platform.

#### Targeted NGS Primer Pools for Pathogen Detection

Primer pools for targeted multiplex RT-PCR (tNGS pathogen panel) were previously designed in collaboration with ThermoFisher Scientific’s AgriSeq Bioinformatics team. These primer pools target many different pathogens in carnivores and have been validated for use in syndromic testing, such as vector-borne [21], respiratory (including SARS-CoV-2 variants) [19,20], and reproductive and neurologic [22] diseases. Conserved gene regions and regions useful for genotyping for multiple pathogen species were identified using published genomic data and compiled into a custom FASTA file. These sequences were processed using a proprietary pipeline to ensure optimal annealing temperatures and to minimize primer–primer interactions. The finalized primers were divided into two optimized pools and paired with a corresponding BED file, both of which were uploaded to the S5 Torrent Server (ThermoFisher Scientific) for sequencing workflow integration. These primer sequences are available from ThermoFisher Scientific [20]. All previous testing with these primer pools was performed on the Ion Torrent sequencing platform according to the manufacturer’s recommendations (ThermoFisher Scientific). Conversion of the assay to the MinION required evaluation of extraction methods and reagents used for reverse transcription and PCR for multiplexing, as well as a different library preparation protocol.

### 2.2. Ruggedness Testing

#### 2.2.1. Extraction Method Comparison

To assess the ruggedness of the tNGS assay using the ONT sequencing method and vulnerability to extraction methods, four nucleic acid extraction kits were compared for their impact on downstream ONT sequencing results. Kits evaluated included column-based extraction method, the DNeasy Blood & Tissue Kit (Qiagen, Venlo, the Netherlands), and three automated protocols: the MagMAX CORE Nucleic Acid Extraction Kit (ThermoFisher Scientific), the MagMAX Pathogen RNA/DNA Kit (ThermoFisher Scientific), and the IndiMag Pathogen Kit (Indical, Leipzig, Germany). No pretreatment was applied to the samples prior to extraction. Automated protocols were executed on a KingFisher Flex Purification System (ThermoFisher Scientific) according to the manufacturer’s instructions.

Six different samples were used for the evaluation, representing tissue pools and oropharyngeal/rectal swabs from three bobcats, two raccoons, and a skunk. Samples were stored at −80 °C until processed for use. For the tissues, samples were cut into small pieces and then mixed with PBS (approximately 1:1) and macerated (Stomacher^®^ 80 Biomaster, Seward Ltd., Worthing, UK). Supernatant was used for the extraction process. Swabs were collected in 3 mL PBS and were vortexed prior to use of the supernatant for extraction. Organisms in the samples included *Canine parvovirus 2a*, *Babesia* sp., *E. coli*, hemotropic *Mycoplasma* sp., and *Clostridium perfringens*, based on previous testing using the tNGS assay on the Ion Torrent System. An equal amount of sample (200 µL) was tested with each kit and nucleic acids were eluted in 90 µL or 100 µL, depending on the kit instructions. Six samples per run were sequenced on a Flongle using the MinION Mk1C (Oxford Nanopore Technologies, Oxford, UK) as described below. For data analysis, reads were demultiplexed by barcode and filtered based on quality metrics (Q score ≥ 9) to improve variance calling and pathogen typing. For each barcode, total and passed bases (Mb), total and passed reads (k), and percentage of passed reads were calculated. Per-kit performance was summarized by averaging values across all six associated barcodes. In addition, run-level statistics including total reads generated, passed reads, estimated bases, N50 read length, and percent base passing thresholds were computed for each extraction kit.

#### 2.2.2. Reverse Transcription PCR

Reverse transcription was performed for all samples prior to targeted amplification. Two reverse transcription kits were evaluated for cDNA synthesis: the NGS Reverse Transcription Kit (ThermoFisher Scientific), used in established Ion Torrent workflows, and the Luna^®^Script RT SuperMix Kit (NEB, Ipswich, MA, USA). Both were used according to the manufacturer’s protocol. For the NGS Reverse Transcription Kit, 7 µL of nucleic acids were combined with 2 µL of buffer and 1 µL of enzyme for a 10 µL total volume. RT conditions were 25 °C for 10 min, 50 °C for 10 min, and 85 °C for 5 min. For Luna^®^Script, 14 µL of nucleic acids was combined with 4 µL of Luna Script RT SuperMix and 2 µL of nuclease-free water in a final 20 µL reaction volume. RT cycling conditions were: 25 °C for 2 min, 55 °C for 10 min, and 95 °C for 1 min. cDNA generated from both kits was used directly in downstream PCR reactions.

#### 2.2.3. PCR Optimization and Polymerase Comparisons

PCR amplification was conducted using multiple enzyme kits to evaluate performance across different workflows, by evaluating the ability to detect SARS-CoV-2 with the tNGS pathogen panel. The Platinum Taq DNA Polymerase (ThermoFisher Scientific) was used with both RT kits. Each 25 µL PCR contained 5 µL of 5X primer pool mix and 10 µL of cDNA, with separate reactions for each primer pool. Cycling conditions were initial denaturation at 94 °C for 2 min, followed by 35 cycles of 94 °C for 30 s and 60 °C for 4 min.

Additionally, cDNA generated from the Luna^®^Script kit was used in PCRs employing Q5^®^ Hot Start High-Fidelity 2X Master Mix (NEB). Each 25 µL reaction consisted of 12.5 µL of Q5 Master Mix, 5 µL of 5X primer pool (1 or 2), and 7.5 µL of cDNA. Thermal cycling was carried out under the following conditions: 98 °C for 30 s, and 35 cycles of 98 °C for 10 s and 60 °C for 4 min.

The NGS Reverse Transcription Kit was also evaluated in combination with Phusion^®^ Hot Start II High-Fidelity DNA Polymerase (ThermoFisher Scientific) for high-accuracy DNA amplification. Each 25 µL reaction included 4 µL of 5X HF Buffer, 0.4 µL of 10 mM dNTPs, 4 µL of primer pool (1 or 2), 0.2 µL of enzyme, 10 µL of cDNA, and 1.4 µL of nuclease-free water. Cycling conditions mirrored those used for the Q5^®^ Hot Start High-Fidelity 2X Master Mix.

### 2.3. Library Preparation and Sequencing

#### 2.3.1. ONT Method

Library preparation for the ONT was performed with the Native Barcode Ligation Kit (Oxford Nanopore Technologies, Oxford, UK). The methodology used in this study is thoroughly described at (https://www.protocols.io/view/oxford-nanopore-targeted-sequencing-assay-for-flon-3byl46ewrgo5/v2, accessed on 23 December 2025) in Protocols.io [23]. The following is a brief overview of the protocol. For the combined protocol that included both the tNGS pathogen panel and the SARS-CoV-2 ARTIC V5.3.2 NCOV-2019 Panel (IDT) (ARTIC) panel, the extracted nucleic acids were used as a template for four independent reverse transcription reactions, two designated for the ARTIC panel and two for the tNGS pathogen panel. The resulting cDNAs were subsequently used in four separate multiplex PCRs: two ARTIC PCRs representing primer Pools 1 and 2, and two tNGS pathogen PCRs representing primer Pools 1 and 2. Platinum Taq DNA Polymerase was used for amplification using the previously described thermocycling conditions. Afterwards, all the PCR’s products were pooled, then bead-purified (AMPure XP) and quantified by a Qubit 4 (ThermoFisher Scientific) with the Qubit dsDNA HS assay (ThermoFisher Scientific). Both panels were also tested individually to determine assay sensitivities for each on the ONT. The purified pooled PCR products were end-prepped using the NEBNext Ultra II End Repair/dA-tailing Module (NEB). Samples were then purified with AmPure XP Beads (Beckman Coulter, Brea, CA, USA) and 7.5 µL purified end-prepped DNA was ligated to a barcode using the NEB Blunt/TA Ligase Master Mix (NEB). Barcoded samples were then pooled (up to six samples per pool) and purified with the AmPure XP Beads, and 30 µL of pooled, purified sample DNA was ligated to the Native Adaptor with the NEBNext Quick Ligation Module (NEB). Purification with AmPure XP Beads was performed again, but Short Fragment Buffer (SFB) was used in place of ethanol to wash the beads. The purified library DNA was quantified with a Qubit and 20 fmol of DNA in 5 µL of Elution Buffer (EB) was loaded onto the Flongle flow cell (Oxford Nanopore Technologies, Oxford, UK) for sequencing.

Sequencing was performed with the MinION Mk1C (ONT) instrument for 24 h. The nanopore raw Pod5 files were base-called with a high-accuracy algorithm to generate FastQ files, which were demultiplexed and trimmed using Dorado 7.1.4 within the MinKNOW software (v24.11.8). Reads with a minimum quality of 9 were used for analysis. The data was analyzed using Geneious Prime 2025.0.3 (https://www.geneious.com/prime, accessed on 23 December 2025). Minimap2 (ver 2.24, data type Oxford Nanopore) was used to map reads to a reference FASTA file that was generated to contain the targeted sequences of the pathogens included in the assay, based on the design of the primer pools. Reads smaller than 100 bases were ignored. Sequences were evaluated with BLAST (https://blast.ncbi.nlm.nih.gov/Blast.cgi, accessed on 23 December 2025) to confirm the results. For SARS-CoV-2 genome analysis, Minimap2 was used to map the sequencing reads to the Wuhan SARS-CoV-2 sequence (NC_045512.2, MN908947.3) in Geneious Prime. The consensus sequence was used to determine the variant type with Nextclade SARS-CoV-2 (XBB data set, updated 17 July 2024). The genome coverage was determined using CZID (https://czid.org/) using the fastQ files obtained from the MinION.

#### 2.3.2. Ion Torrent Method

The cDNA was prepared with the NGS RT kit as previously described. Multiplex PCR (tNGS pathogen panel), automated library preparation, and chip loading were performed in-house using the Ion Chef™ Instrument (ThermoFisher Scientific), followed by sequencing on the Ion GeneStudio™ S5 System (ThermoFisher Scientific) following our previously published protocol using a 530 chip [20].

### 2.4. Inclusivity

Each Omicron subvariant (Table 1) was spiked undiluted 1:1 into nucleic acids obtained from six different wildlife species (two subvariants used per wildlife sample) that tested negative for SARS-CoV-2 by the RT-PCR. This was the same wildlife sample set used for the ruggedness testing. These contrived positive samples were tested with the targeted NGS pathogen panel on the Ion Torrent platform (Supplementary File S1). Older SARS-CoV-2 variants were previously validated with this panel [20].

### 2.5. Limit of Detection/Analytical Sensitivity

Ten-fold serial dilutions of six of the SARS-CoV-2–positive nucleic acids obtained from the Indiana Public Health Laboratory were prepared and spiked into nucleic acids extracted from wildlife samples that had tested RT-PCR negative for SARS-CoV-2. This resulted in contrived positive samples with Ct values ranging from 27–38. Spiked samples were tested in parallel with the RT-PCR and the tNGS pathogen assay on the ONT platform. NGS testing was performed with Omicron subvariants on three different days (two subvariants per run).

A subset of the contrived positive samples with dilutions in the range of Ct values from 27–31, representing the same six Omicron subvariants, were further tested using the SARS-CoV-2 ARTIC V5.3.2 NCOV-2019 primer sets. The targeted multiplex RT-PCR was performed with the NGS RT and Platinum Taq as previously described, except the primer pool volume was 4 µL instead of 5 µL (primer pool dilutions made according to manufacturer’s recommendations), and the master mix was adjusted accordingly. Library prep and sequencing were performed as previously described for ONT sequencing. Contrived positive samples were also tested with the combined protocol on the MinION, as previously described, using the NGS RT and Platinum Taq methods for targeting.

### 2.6. Analytical Specificity

The analytical specificity of the assay primers was further evaluated in silico by comparing the sequencing read results for specific pathogens using nucleotide BLAST (https://blast.ncbi.nlm.nih.gov/Blast.cgi, accessed on 23 December 2025) to confirm target identity.

### 2.7. Diagnostic Sensitivity and Specificity

In total, 60 of the 161 wildlife samples were included in this evaluation. Thirty wildlife samples that were known to be negative for SARS-CoV-2, based on testing with an approved RT-PCR (TaqPath COVID-19 PCR Kit, ThermoFisher Scientific), were sequenced using the primer sets for targeting pathogens with both the Ion Torrent method (considered the gold standard test) and an ONT method. Additionally, another set of thirty negative wildlife samples were spiked with Omicron subvariants with Ct values ranging from 16.61 to 37.41, based on the RT-PCR results for sensitivity testing. A comparison between the Ion Torrent and the ONT platforms was performed to evaluate the targeted detection of SARS-CoV-2 and pathogens other than SARS-CoV-2 between the two methods.

### 2.8. Wildlife Surveillance

The combined tNGS assay was applied to a separate set of 95 wildlife samples collected for routine surveillance purposes. These were distinct from the samples used in analytical sensitivity and extraction-method experiments. This was a pilot study using the combined SARS-CoV-2 ARTIC and pathogen-specific primer panels to enable simultaneous detection of SARS-CoV-2 and other viral, bacterial, and fungal agents of veterinary and zoonotic relevance.

### 2.9. Statistical Analysis

Statistical analyses performed to test significant differences among the extraction kits were conducted in R (version 4.4.2). For each outcome variable total bases (Mb), percent passed reads, and percent passed bases one-way analysis of variance (ANOVA) was performed. When significant differences were found (*p* < 0.05), Tukey’s Honest Significant Difference (HSD) post hoc tests were used to identify pairwise differences. Group means were summarized, and statistical groupings were assigned based on significance. The performance of the four extraction kits was further compared using parvovirus detection counts, which were evaluated for normality (Shapiro–Wilk test) and homogeneity of variances (Levene’s test) prior to pairwise comparisons. All statistical analyses and visualizations were performed in R using base R functions along with the ggplot2, dplyr, and multcomp packages.

## 3. Results

### 3.1. Ruggedness Testing Results

Sequencing output and read quality were evaluated across four different extraction kits, revealing that while there was no statistically significant difference in the total amount of sequencing data generated (F (3,20) = 1.05, *p* = 0.394), notable differences were observed in read quality metrics (Table 2). All kits produced comparable sequencing throughput, with the Column and MagMAX CORE Nucleic Acid Extraction kits yielding slightly higher average base outputs (~130 Mb) and the IndiMag Pathogen and MagMAX Pathogen RNA/DNA kits yielding slightly lower averages (~98 Mb) (Table 2). Analysis of read quality showed significant variation between kits. ANOVA indicated a strong effect of the extraction kit on both the percentage of passed reads (F (3,20) = 16.97, *p* = 1.01 × 10^−5^) and passed bases (F (3,20) = 14.03, *p* = 3.74 × 10^−5^). Post hoc Tukey HSD analysis identified the MagMAX Pathogen RNA/DNA kit as significantly outperforming the other three kits in both metrics (*p* < 0.001), with mean values of 91.9% passed reads and 88.4% passed bases, compared to 74.8–76.6% for the Column, MagMAX CORE Nucleic Acid Extraction kit, and IndiMag Pathogen kits (Table 2). Based on Tukey groupings, the MagMAX Pathogen RNA/DNA kit was assigned to group “a” (significantly better), while the other three kits were grouped as “b” (not significantly different from one another). These findings indicate that while all extraction kits can generate comparable volumes of sequencing data, the MagMAX Pathogen RNA/DNA kit offers superior read and base quality, making it the preferred choice for workflows requiring high-quality sequencing.

To further compare kit performance using a biological endpoint, parvovirus detection counts were analyzed (Table 3). Shapiro–Wilk tests indicated that data from three kits (DNeasy, IndiMag, and Pathogen) deviated from normality (*p* < 0.05), whereas data from the MagMAX CORE kit were approximately normal (*p* > 0.05). Levene’s test confirmed homogeneity of variances among kits (F (3,20) = 1.07, *p* = 0.383), supporting the use of parametric analyses. Repeated measures ANOVA revealed no statistically significant differences among kits (*p* > 0.05). Post hoc pairwise t-tests with Bonferroni correction likewise detected no significant pairwise differences (Table 4).

PCR amplification using Platinum Taq with either RT kit (Luna or NGS RT) was found to be equivalent in yield and specificity, validating both workflows for targeted pathogen amplification in wildlife samples. The other Taq polymerases (Phusion^®^ Hot Start II High-Fidelity DNA Polymerase and the Q5^®^ Hot Start High-Fidelity 2X Master Mix) failed to amplify all the samples to detectable levels.

### 3.2. Limit of Detection/Analytical Sensitivity, Specificity, and Inclusivity

We determined that the approximate limit of detection LOD, when compared to RT-PCR, was a Ct of 34 for detection of SARS-CoV-2 using the tNGS pathogen assay on the ONT. All Omicron subvariants tested for inclusivity were detectable with the tNGS assay. Based on the LOD testing, SARS-CoV-2 could be detected with the ONT method at the Ct value equivalent of 34. When testing the SARS-CoV-2 tNGS assay alone, we were able to sequence genomes with ≥99% coverage of the genome and were accurately able to type the subvariants up to a virus load consistent with a Ct value of 31, based on results provided by the Indiana Department of Health. However, sequence depth across the full genomes fell below the required limit of at least 60X for appropriate quality control. Use of six samples per Flongle produced ≥ 1 × 10^6^ reads (≥100,000 reads per sample).

### 3.3. Diagnostic Sensitivity and Specificity

Targeted NGS was performed using both the Ion Torrent Method and the ONT method. The detection of SARS-CoV-2 between the ONT method and the RT-PCR was compared. The diagnostic sensitivity and specificity for the detection of SARS-CoV-2 for the ONT method were calculated and compared to the results from the Ion Torrent method. Evaluation of detection of SARS-CoV-2 with the ONT method compared to the RT-PCR, three SARS-CoV-2 spiked samples were missed by the ONT tNGS pathogen assay (Table 5). The Ct values for these samples were 36–37, which is considered above the relative detection limit of 34 for this method using ONT. Cohen’s kappa was also calculated to determine agreement between the two methods, beyond what would be expected by chance. This resulted in a sensitivity of 96.7% and a specificity of 100%, accuracy of 98.2% for SARS-CoV-2 for the tNGS methods when comparing the Ion Torrent and the ONT methods (Table 5). Cohen’s kappa was 0.968, suggesting almost perfect agreement between the two platforms. For the other pathogens in the assay, in general, the ONT method was better for detecting *E. coli eae* and *Campylobacter jejuni* (Table 6). Based on sequence comparisons, several samples showed nucleotide differences between the amplicon sequences obtained from the sample set and the sequences represented in the reference FASTA used for mapping. Despite these mismatches, the MinION workflow consistently generated full amplicon coverage, whereas the Ion Torrent platform demonstrated reduced coverage or amplicon dropout in the same regions. This might be due to differences in the mapping. For the Ion Torrent data, the suite software was used to map the sequences to the reference FASTA file. Minimap2 was used to map the ONT reads to the same reference file. There was greater tolerance for sequence divergence from the reference sequences, at least for *E. coli eae* and *C. jejuni*, when running the samples with the ONT and associated data analysis protocol. However, for sample #5, the sensitivity was higher with the Ion Torrent method for *C. perfringens cpe* and *epsilon*. When using a cut-off of 10 reads to call a positive result, we would miss *C. perfringens cpe* (two reads) and *epsilon toxin* (three reads) in sample #5 with the ONT method. This is due to the reduced number of reads we obtained with the ONT method when using six samples per Flongle, compared to the Ion Torrent method. We proved this by re-testing samples 13–18, using only three samples per Flongle instead of six (Table 6). We were able to reproduce the results obtained with the Ion Torrent with three samples, but not with the original six on the Flongle. In addition to the spiked SARS-CoV-2, the targeted method detected several zoonotic pathogens within this sample set (Table 6). For the otters (negative SARSCoV-2 sample set for specificity testing), normal flora was detected, in addition to some pathogens, including CPV and CDV. Several of these river otters had detectable amounts of *Chlamydia* sp., and Candidatus *Mycoplasma* sp. were detected in some of these otters, but the species could not be determined based on the targeted region of the genome. (Table 7).

To evaluate sequencing depth and variant resolution with the combined assay, contrived positive samples were analyzed. Presence of a large number of other pathogens reduced the ability to fully sequence the genome. However, a representative sample (#28 from the diagnostic sensitivity testing) containing Canine distemper virus (Ct 17) spiked with SARS-CoV-2 (Ct ≈ 24) produced robust sequencing performance, achieving 293X average genome coverage, and simultaneously detecting CDV with over 200,000 reads. Variant identification remained reliable up to approximately Ct 24; above Ct 31, coverage became insufficient for definitive variant typing, despite running three samples per Flongle cell.

### 3.4. Wildlife Surveillance

The 95 samples tested by the combination assay were all negative for SARS-CoV-2. This was confirmed by negative results for each sample with the SARS-CoV-2 RT-PCR assay.

## 4. Discussion

Effective pathogen surveillance in wildlife is critical for early detection of emerging zoonoses, yet molecular approaches based on short-read sequencing often face challenges related to scalability, cost, and field deployment. In this work, we evaluated the ONT as a potentially portable, cost-effective method for tNGS. The platform’s portability and relatively low operating costs, particularly when paired with the Flongle flow cell, make it well-suited for applications in resource-limited environments. Using SARS-CoV-2 as a proof-of-concept pathogen, we successfully adapted a laboratory-developed tNGS pathogen panel, originally designed for short-read sequencing, for use on the ONT. This panel targets multiple pathogens relevant to carnivores, including SARS-CoV-2, thereby enabling simultaneous detection of both endemic and emerging threats within a single assay. Such multiplexing improves efficiency, reduces response times, and demonstrates the potential of ONT-based tNGS for wildlife and One Health surveillance.

Although all wildlife samples tested were negative for SARS-CoV-2, several zoonotic pathogens were detected, including *Salmonella* spp., *Listeria monocytogenes*, *Campylobacter jejuni*, *Clostridium perfringens* type F, STEC, and *Toxoplasma gondii*. These results highlight that limiting surveillance to a single pathogen, such as SARS-CoV-2, using PCR, risks overlooking other important agents with potential public health and ecological impact. Notably, we also identified *Cytauxzoon felis* in a bobcat, consistent with the role of bobcats (*Lynx rufus*) as natural reservoirs of this parasite. While domestic cats often experience severe, frequently fatal cytauxzoonosis [24], bobcats generally harbor subclinical infections that support long-term parasite maintenance and transmission [25]. The confirmation of *C. felis* using targeted NGS underscores both the value of molecular tools for wildlife hosts lacking overt clinical signs and the importance of surveillance at the wildlife–domestic interface [26,27], where spillover may occur via tick vectors such as *Amblyomma americanum* and *Dermacentor variabilis* [28]. Furthermore, the detection of *C. felis* in a bobcat sample by both the MinION (59,900 reads) and Ion Torrent (43,512 reads) platforms demonstrates strong cross-platform concordance and further validates the reliability of the adapted tNGS workflow for parasite detection in wildlife. Importantly, the reproducibility of *C. felis* detection across two independent sequencing platforms reduces the likelihood of spurious assignment and provides confidence that the finding represents a true infection. These results also illustrate the utility of integrating nanopore sequencing into wildlife pathogen surveillance: not only does it provide results consistent with established short-read platforms, but its portability and lower per-run cost enable broader application in field and diagnostic contexts. Several otter samples were also tested, and the organisms detected in these cases were largely consistent with normal flora. However, river otters are harboring CDV and CPV and could serve as a source for other animals that can be infected with these pathogens. Additionally, 9/30 otters had detectable *Chlamydia* sp., but the significance of this is unknown. Two of these North American river otters (*Lontra canadensis*) had detectable Candidatus *Mycoplasma* sp. The area targeted based on the primer design could not speciate this organism; however, there was a new species of Candidatus *Mycoplasma* described recently in the Neotropical otter (*Lontra longicaudis*) [29]. Again, the significance of this finding is unknown.

When benchmarked against the Ion Torrent Chef/S5, agreement between the two sequencing platforms was high for most pathogens, though the MinION performed better for detecting *E. coli eae* and *Campylobacter jejuni*. Sequence comparisons revealed variation between reads from our samples and reference FASTA sequences, suggesting that the MinION workflow with associated data analyses more tolerant of sequence variation than the Ion Torrent workflow. Nonetheless, in certain cases (e.g., sample #5), Ion Torrent demonstrated higher sensitivity. Moreover, using a cutoff of 10 reads for positivity, the MinION platform missed *Clostridium perfringens* cpe and epsilon toxin genes, likely due to lower sequencing depth. Retesting samples with reduced loading (three samples per Flongle instead of six samples) restored concordance with Ion Torrent, illustrating that sequencing depth is a critical determinant of sensitivity in NGS assays. The three contrived samples that were not detected by the ONT assay had Ct values of 36–37, which exceed the relative detection limit of approximately Ct 34 established for this MinION workflow, indicating that the missed detections reflect low input rather than lineage-specific primer dropout. These three subvariants were detected with the inclusivity testing.

Importantly, combining primers for pathogen detection with those for SARS-CoV-2 whole-genome sequencing yielded ≥99% genome coverage. The 293X genome coverage obtained for sample #28 greatly exceeds the ~60X depth typically required for confident SARS-CoV-2 variant assignment, demonstrating that the combined assay can support accurate genotyping even in the presence of high-abundance co-infecting pathogens. Running three samples per Flongle generated sufficient reads to achieve whole-genome sequencing of SARS-CoV-2 down to a Ct of 24, while simultaneously detecting additional pathogens. Reducing to three samples per Flongle further improved sequencing depth and quality, though at increased per-sample cost. These findings confirm that multiplexed panels integrating both pathogen detection and viral WGS are feasible on nanopore platforms.

The extraction method also influenced sequencing performance. While all four kits tested produced comparable total yields, read quality varied significantly. The MagMAX Pathogen RNA/DNA kit consistently outperformed others in the proportion of passed reads and bases, metrics that are critical for downstream analyses such as variant calling, taxonomic assignment, and detection of low-abundance targets [30]. This is likely due to the four washing steps associated with this extraction method, which is more than the other methods. In contrast, the Column, MagMAX CORE Nucleic Acid Extraction, and IndiMag Pathogen kits yielded similar but lower-quality data, making them more suitable where throughput is prioritized over accuracy. These observations emphasize that, beyond yield, read quality should be a key consideration when selecting extraction protocols for sensitive sequencing applications. However, it is important to note that there was no appreciable difference between kits and the ability to detect the various pathogens detected in the samples, including SARS-CoV-2. This finding indicates that for routine targeted detection workflows, these extraction kits can be used interchangeably. Consistent with this observation, comparative analysis of parvovirus detection counts confirmed that all four extraction kits performed comparably under similar testing conditions, supporting their interchangeable use and providing flexibility for laboratories based on available resources.

Finally, this study demonstrates that nanopore sequencing can be effectively implemented on the Flongle as a proof-of-concept platform. Although the Flongle contains far fewer active pores (~60–100) and produces lower yields (1–2 Gb) compared to standard MinION flow cells (>800 pores; 10–20 Gb), the data quality and recovery of target sequences were consistent. The error profiles, Q-score distributions, and demultiplexing performance were comparable to larger flow cells, supporting extrapolation of our findings. Scaling to full-sized flow cells would be expected to increase throughput, enhance sensitivity for low-abundance pathogens, and reduce stochastic dropouts, thereby strengthening diagnostic reliability. Larger flow cells would also permit simultaneous processing of more samples and technical replicates, improving robustness and cost-effectiveness in routine surveillance. Collectively, these findings establish a foundation for scalable, low-cost, ONT-based tNGS workflows with clear applications for wildlife disease monitoring, public health, and conservation biology.

## 5. Conclusions

The adapted tNGS panel on the MinION platform using a Flongle demonstrated the ability to detect SARS-CoV-2 and other pathogens from wildlife samples at a lower cost than can be done for low numbers of samples on the Ion Torrent platform.

## Figures and Tables

**Table 1:** Ct Values and Variant Classification for SARS-CoV-2 Positive Samples.

Sample	Clade	Lineage	Original Ct Value
C23001609	BA.5.1	BA.5.1	16.3
C23002860	XBB.1.5	XBB.1.5.7	21.5
C23002864	BA.2.75.3.1.1.1.1	XBX	16.9
C23006521	XBB.1.5	XBB.1	16.3
C23015807	BA.5.3.1.1.1.1.1	BQ.1	20.6
C23015808	XBB.1.5	XBB.1.5	27.3
C23046902	XBB.1.9.1	FL.1.5.1	22.5
C23047107	XBB.1.9.1	XBB.1.16	16.2
C23048464	XBB.1.9.1	EG.5.1.1	15.8
C23054397	XBB.1	XBB.1.16.6	17.4
C23063232	XBB.1	HK.3	16.2
C23065345	B.1.1.529	BA.2	15.7

**Table 2:** Comparison of Sequencing Metrics Across DNA Extraction Kits.

Kit	Reads Generated (M)	Passed Reads (M)	Failed Reads (M)	Estimated Bases (Mb)	Passed Bases (Mb)	Failed Bases (Mb)	N50 (bp)	Run Duration (hrs)	% Passed Reads	% Passed Bases
DNeasy	2.1	1.84	0.305	936.99	717.67	126.3	395	24	87.6	76.6
MagMAX CORE	2.04	1.71	0.363	936.18	715.47	164.42	398	24	83.8	76.4
IndiMag Pathogen	1.35	1.19	0.187	719.34	538.24	94.4	407	24	88.1	74.8
MagMAX Pathogen	1.36	1.25	0.121	644.8	569.9	59.2	406	24	91.9	88.4

**Table 3:** Comparison of four extraction kits for use with tNGS. 200 µL was used for each sample for each kit. Elution was in 90 or 100 µL of elution buffer, depending on the kit used.

Samples	Dneasy Blood&Tissue Kit (Column)	MagMax^TM^ CORE Nucleic Acid extraction Kit	IndiMag Pathogen Kit	MagMax Pathogen RNA/DNA Kit
Raccoon A	CPV: 89 reads Hemotropic *Mycoplasma* sp.: 10 reads	CPV: 53 reads Hemotropic *Mycoplasma* sp.: 14 reads	CPV: 947 reads Hemotropic *Mycoplasma* sp.: 75 reads	CPV: 27 reads Hemotropic *Mycoplasma* sp.: 29 reads
Raccoon B	CPV: 78 reads *Babesia* sp.: 13 reads	CPV: 88 reads *Babesia* sp.: 100 reads *E. coli* eae: 4 reads	CPV: 450 reads *Babesia* sp.: 100 reads *E. coli* eae: 35 reads	CPV: 72 reads *Babesia* sp.: 200 reads *E. coli* eae: 4 reads
Bobcat A	CPV: 208 reads	CPV: 9521 reads *Clostridium perfringens*: 16 reads	CPV: 10,732 reads *Clostridium perfringens*: 11 reads	CPV: 222 reads *Clostridium perfringens*: 5 reads
Bobcat B	CPV: 25,596 reads *E. coli* eae: 16 reads *Clostridium perfringens*: 19 reads	CPV: 40,217 reads	CPV: 182,531 reads	CPV: 31,146 reads
Otter A	CPV: 108 reads	CPV: 135 reads	CPV: 984 reads	CPV: 31 reads
Skunk A	CPV: 91 reads	CPV: 101 reads	CPV: 24,711 reads	CPV: 74 reads

**Table 4:** Statistical analysis of four different extraction kits (*p* < 0.05 was considered statistically significant).

Kits Compared	DNeasy	IndiMag	MagMax CORE	Pathogen
DNeasy	-	1	0.49	1
IndiMag	1	-	0.88	1
MagMax CORE	0.49	0.88	-	1
Pathogen	1	1	1	-

**Table 5:** 2 × 2 table displaying comparative results for SARS-CoV-2 tested by the ONT and Ion Torrent methods, based on contrived positive samples and negative mesocarnivore samples.

	Ion Torrent Method	
ONT Method	Positive	Negative
**Positive**	29	0
**Negative**	1	30

Positive Percent Agreement 96.7% (95% CI 82.78–99.92), Negative Percent Agreement 100.00% (91.40–100.00), Accuracy, (total percent agreement with 95% CI) 98.2% (92.40–99.96). Cohen’s Kappa—0.968, almost perfect agreement.

**Table 6:** Diagnostic Sensitivity testing with contrived SARS-CoV-2 positive samples (based on NAAT results). A comparison of the targeted assay using the Ion Torrent and associated library prep and data analysis vs. the MinION and its associated library prep and data analysis was performed.

	Sample Name	Spiked SARS-CoV-2 Lineage	Ion Torrent	MinION
1	A25-8520-1 (Raccoon feces) Animal Rescue in WA, recovered from CDV	BA.5.1 Ct 28.46	*E. coli cnf1*, *C. perfringens alpha*, SARS-CoV-2, **no *eae o* and no *C. jejuni***	*E. coli cnf1*, *eae*, *C. perfringens alpha*, *C. jejuni*, SARS-CoV-2
2	A25-8520-2 (Raccoon feces) Animal Rescue in WA, recovered from CDV	BA.5.1 Ct 28.46	*C. perfringens alpha*, *E. coli cnf*, SARS-CoV-2, *Listeria monocytogenes*, **no *eae* and no *C. jejuni***	*C. perfringens alpha*, *E. coli cnf*, *eae*, SARS-CoV-2, *Listeria monocytogenes monocytogenes*, *C. jejuni*
3	A25-8520-3 (Raccoon feces) Animal Rescue in WA, recovered from CDV	BA.5.1 Ct. 28.46	*C. perfringens alpha*, SARS-CoV-2, *E. coli cnf*, *eae*, *Listeria monocytogenes*, *C. jejuni, Lawsonia intracellularis*, **no *C. jejuni***	*C. perfringens alpha*, *E. coli cnf1*, *eae*, *Listeria monocytogenes*, *C. jejuni*, SARS-CoV-2, *Lawsonia intracellularis*
4	A25-8520-4 (Raccoon feces) Animal Rescue in WA, recovered from CDV	XBB.1 Ct 24.52	*E. coli cnf*, *eae*, *C. perfringens alpha*, SARS-CoV-2, *Listeria monocytogenes*, *C. jejuni*	*C. perfringens alpha*, *E. coli cnf1,2*, *eae*, SARS-CoV-2, *C. jejuni*
5	A25-8520-5 (Raccoon feces) Animal Rescue in WA, recovered from CDV	XBB.1 Ct 24.52	*C. perfringens alpha*, *cpe (85 reads) epsilon (13 reads), E. coli cnf1*, *eae*, SARS-CoV-2, *Listeria monocytogenes*, *C. jejuni*	*C. perfringens alpha*, ***cpe (2 reads)-neg***, ***epsilon (3 reads)-neg***, *E. coli cnf1*, *eae*, *C. jejuni*, SARS-CoV-2, *Listeria monocytogenes*
6	A25-8520-6 (Raccoon feces) Animal Rescue in WA, recovered from CDV	XBB.1.16 Ct 18	SARS-CoV-2, *C. perfringens alpha*, *E. coli cnf*, *eae*, *C. jejuni*, *Chlamydia* sp.	SARS-CoV-2, *C. perfringens alpha*, *E. coli cnf1*, *eae*, *C. jejuni*, *Chlamydia* sp.
7	Bobcat 2 Tissue pool	XBB.1.16 Ct 22.21	*Cytauxzoon felis*, *C. perfringens alpha*, *cpe*, CPV 2a or panleukopenia *Salmonella* sp., *E. coli stx2*, SARS-CoV-2, **no *C. jejuni***	*Cytauxzoon felis*, *C. perfringens alpha*, *cpe*, SARS-CoV-2, CPV 2a/panleukopenia, *C. jejuni*, *Salmonella* sp., *E. coli stx2*
8	Otter 739 Tissue pool	XBB.1.16 Ct 22.21	CPV, SARS-CoV-2, *C. perfringens alpha*	CPV, SARS-CoV-2, *C. perfringens alpha*
9	Otter 744 Tissue pool	EG.5.1.1 Ct 27	CPV, *C. perfringens alpha*, *netE*, *E. coli eae*, *cnf1*, *haemotropic Mycoplasma* sp.; SARS-CoV-2	SARS-CoV-2, *E. coli eae*, *cnf1*, *C. perfringens alpha*, *net E*, CPV, *haemotropic Mycoplasma* sp.
10	Bobcat 3 Tissue pool	EG.5.1.1 Ct 27	*C. perfringens alpha*, *beta 2*, *net E*, *cpe*, *CPV*, *Toxoplasma gondii*, *E. coli cnf1*, SARS-CoV-2	*CPV*, *C. perfringens alpha*, *cpe*, *beta 2*, *net E*, SARS-CoV-2, *E. coli cnf1*, *Toxoplasma gondii*
11	Raccoon 7-Swab-rectal and oropharynx	48464-2EG.5.1.1 Ct 27	CPV, *C. perfringens alpha*, *beta 2*, *cpe*, *net E*, *cpe*, *E. coli cnf1*, SARS-CoV-2, **no *C. jejuni*** and ***E. coli eae***	*C. jejuni*, SARS-CoV-2, CPV, *C. perfringens alpha*, *eae*, *net E*, *beta2*, *cpe*, *E. coli cnf1*
12	Bobcat 6 small intestine and colon	FL.1.5.1 Ct 31.31	*C. perfringens alpha*, *beta2*, *cpe*, *net E*, CPV, SARS-CoV-2-, *E. coli cnf1*, *eae*	*C. perfringens alpha*, *cpe*, *beta2*, *net E*, CPV, SARS-CoV-2, *E. coli cnf1*, *eae*
13	Bobcat 10 Tissue pool	XBX Ct 25.5	*C. perfringens alpha*, *netE*, *beta2*, CPV, SARS-CoV-2, *Giardia intestinalis*, *E. coli hly*	*C. perfringens alpha* only **(repeat with only 3 samples-*C. perfringens alpha***, ***cpe***, ***netE***, ***beta2***, **CPV**, **SARS-CoV-2**, ***Giardia intestinalis***, ***E. coli hly***, ***eae***, ***cnf1*)**
14	Bobcat 7 Tissue pool	XBX Ct 25.5	SARS-CoV-2, *E. coli cnf1*, *C. perfringens alpha*	No pathogens detected **(repeat with only 3 samples-SARS-CoV-2**, ***E. coli cnf1***, ***C. perfringens alpha*)**
15	Bobcat 9 Tissue pool	XBX Ct 25.5	CPV, SARS-CoV-2	CPV only **(repeat with only 3 samples-CPV**, **SARS-CoV-2)**
16	Raccoon 6 Swab- rectal and oropharynx	XBB.1.16 Ct 22.21	SARS-CoV-2, *E. coli cnf1*, *eae*, *C. perfringens alpha*, *C. jejuni*	SARS-CoV-*2*, *E. coli eae*, *cnf1* **(repeat with only 3 samples-SARS-CoV-2**, ***E. coli cnf1***, ***eae***, ***C. perfringens alpha***, ***C. jejuni*)**
17	Raccoon 3- Swab- rectal and oropharynx	XBB.1.16 Ct 22.21	SARS-CoV-2, *E. coli cnf1*, *C. perfringens alpha*, CPV	SARS-CoV-2 only **(repeat only 3 samples-SARS-CoV-2**, ***E. coli cnf1***, ***C. perfringens alpha***, **CPV)**
18	Raccoon 1- Swab- rectal and oropharynx	XBB.1.9.1 Ct 22.21	SARS-CoV-2, *E. coli cnf1*, CPV, *C. perfringens alpha*	SARS-CoV-2, *E. coli cnf1* **(repeat only 3 samples SARS-CoV-2**, ***E. coli cnf1***, ***C. perfringens alpha***, ***cpe***, **CPV)**
19	Racoon 5- Swab- rectal and oropharynx	FL.1.5.1 Ct 28.42	SARS-CoV-2, *C. perfringens alpha*, *haemotropic Mycoplasma* sp., *E. coli cnf1*, CPV	*C. perfringens alpha*, SARS-CoV-2, CPV, *E. coli cnf1*, *haemotropic Mycoplasma* sp.
20	Bobcat 4 small intestines and colon	FL.1.5.1 Ct 28.42	*C. perfringens alpha*, *net E*, SARS-CoV-2, *E. coli cnf1* **no *C perfringens epsilon or E. coli eae***	*C. perfringens alpha*, *Net E*, *epsilon*, SARS-CoV-2, *E. coli eae*, *cnf1*
21	otter 711 Tissue pool	FL.1.5.1 Ct 28.42	*C. perfringens alpha*, SARS-CoV-2, *E. coli cnf1*, *stx2*	*C. perfringens alpha*, *SARS-CoV-2*, *E. coli cnf1*, *stx2*
22	Raccoon 3- Swab- rectal and oropharynx	FL.1.5.1 Ct 28.42	*E. coli cnf1,2*, *eae*, *C. perfringens alpha*, *cpe*, SARS-CoV-2, *Rickettsia* sp., *Listeria monocytogenes*	*C. perfringens alpha*, *cpe*, SARS-CoV-2, *E. coli crnf2*, *cnf1*, *eae*, *Rickettsia* sp., *Listeria monocytogenes*
23	A25-9371 Sm. intestine/mesenteric lymph node pool (Raccoon-mass mortality event Jasper Co, Dx CPV)	XBX Ct 37.41	CPV 2a, *E. coli cnf1 and 2*, *Campy jejuni*, *C. perfringens alpha*, *cpe*, *Babesia* sp., **no *Salmonella* sp.**, **no SARS-CoV-2**	*Salmonella* sp. (16 reads), CPV *2a*, *E. coli cnf1*, *cnf2*, *C. perfringens alpha*, *cpe*, **no SARS-CoV-2**, *C. jejuni*, *Babesia* sp.
24	A25-9371 lung (Raccoon mass mortality event Jasper Co., Dx CPV)	HK.3 Ct 33.18	*Bordetella* sp., SARS-CoV-2, *Chlamydia* sp., CPV, *E. coli cnf1*	*Bordetella* sp., SARS-CoV-2, *Chlamydia* sp., CPV, *E. coli cnf1*
25	Bobcat 2022 Small intestine and colon	XBB.1.5.7 Ct 29.76	CPV 2a, SARS-CoV-2, *C. perfringens alpha*	CPV 2a, SARS-CoV-2, *C. perfringens alpha*
26	Bobcat 2022 lung	XBX Ct 36.2	CPV 2a, SARS-CoV-2	*CPV 2a*, **no SARS-CoV-2**
27	S23-646 spleen Gray fox, Perry Co.—aggressive, tick infestation, CDV PCR negative	BA.5.1 Ct 28.46	SARS-CoV-2, *Staphylococcus aureus*, *Babesia vulpes*, *Hepatozoon canis*	SARS-CoV-2, *Staphylococcus aureus*, *Babesia vulpes*, *Hepatozoon canis*
28	A24-7980 lung (Raccoon, Neuro signs, Delaware Co, CDV Ct 17)	XBB.1 Ct 24.52	SARS-CoV-2, CDV- AM-5	SARS-CoV-2, CDV AM-5
29	Bobcat 4 lung	XBX Ct 37.41	**no SARS-CoV-2**, *Cytauxzoon felis*, **no *Hepatozoon* sp.**	*C felis*, *Hepatozoon* sp. **no SARS-CoV-2**
30	Bobcat 8 lung	BA.5.1 Ct 28.46	SARS-CoV-2, *Haemotropic-Mycoplasma* sp., *E. coli cnf1*, *Staphyloccus aureus*, *C. perfringens alpha*	*E. coli cnf1*, *Staphyloccus aureus*, *C. perfringens alpha*, SARS-CoV-2, *Haemotrophic Mycoplama* sp.

Bolded text indicates results that were not detected.

**Table 7:** Diagnostic specificity testing of negative SARS-CoV-2 samples (based on NAAT). A comparison of the targeted assay using the Ion Torrent and associated library prep vs. the MinION and its associated library prep was performed.

Number	Sample Name	Ion Torrent	MinION
1	Otters135	*Actinomyces* spp., *C. perfringens alpha*, *E. coli*	*Actinomyces* spp., *C. perfringens alpha*, *E. coli*
2	Otters136	*E. coli*, *Actinomyces* spp., *C. perfringens alpha*	*E. coli*, *Actinomyces* spp., *C. perfringens alpha*
3	Otters137	*Actinomyces* spp., *E. coli*	*Actinomyces* spp., *E. coli*
4	Otters138	*Actinomyces* spp., *C. perfringens alpha*, *E. coli*	*Actinomyces* spp., *C. perfringens alpha*, *E. coli*
5	Otters139	CPV, *Bordetella bronchiseptica*, *Actinomyces* spp.	CPV, *Bordetella bronchiseptica*, *Actinomyces* spp.
6	Otters140	*Actinomyces* spp.	*Actinomyces* spp.
7	Otters141	*Actinomyces* spp., *Candidatus Mycoplasma* sp., CPV, *C. perfringens alpha*	*Actinomyces* spp., *Candidatus Mycoplasma* sp., CPV, *C. perfringens alpha*
8	Otters142	*Actinomyces* sp., CPV, *C. perfringens alpha*	*Actinomyces* spp., CPV, *C. perfringens alpha*
9	Otters 144	*Streptococcus canis*, *Actinomyces* spp.	*Streptococcus canis*, *Actinomyces* spp.
10	Otters 145	*Actinomyces*, *Chlamydia* sp., CDV	*Actinomyces*, *Chlamydia* sp., CDV
11	Otters 146	*Mycoplasma* sp., CPV, *Actinomyces* sp.	*Mycoplasma* sp., CPV, *Actinomyces* sp.
12	Otters 147	CDV, CPV, *Actinomyces*, *E. coli*, *C. perfringens alpha*	CDV, CPV, *Actinomyces*, *E. coli*, *C. perfringens alpha*
13	Otters 148	*Actinomyces* spp.	*Actinomyces* spp.
14	Otters 149	*Actinomyces* sp., *E. coli*	*Actinomyces* sp., *E. coli*
15	Otters 150	*Actinomyces* sp., *C. perfringens alpha*, *E. coli*	*Actinomyces* sp., *C. perfringens alpha*, *E. coli*
16	Otters 151	*Actinomyces* sp., *Mycoplasma* sp.	*Actinomyces* spp., *Mycoplasma* sp.
17	Otters 152	*Actinomyces* sp.	*Actinomyces* spp.
18	Otters 153	*Actinomyces* sp., *Candidatus Mycoplasma* sp., *Chlamydia* sp., CPV, *C. perfringens alpha*, *E. coli*	*Actinomyces* sp., *Candidatus Mycoplasma* sp., *Chlamydia* sp., CPV, *C. perfringens alpha*, *E. coli*
19	Otters 154	*C. perfringens alpha*, *Actinomyces* sp., *E. coli*, CPV, *Streptococcus canis*, *Bordetella* sp.	*C. perfringens alpha*, *Actinomyces* sp., *E. coli*, CPV, *Streptococcus canis*, *Bordetella* sp.
20	Otters 155	*Mycoplasma* sp., *CPV*, *Helicobacter* spp., *Actinomyces* spp.	*Mycoplasma* sp., CPV, *Helicobacter* spp., *Actinomyces* spp.
21	Otters 156	*Actinomyces* spp., CPV, *Mycoplasma* sp., *Chlamydia*, *Helicobacter* spp.	*Actinomyces* spp., CPV, *Mycoplasma* sp., *Chlamydia*, *Helicobacter* spp.
22	Otters 157	*Actinomyces*, *Helicobacter* spp., *Chlamydia*	*Actinomyces*, *Helicobacter* spp., *Chlamydia*
23	Otters 158	*Actinomyces* spp., CPV, *Mycoplasma* sp., *Chlamydia*, *C. perfringens alpha*, *Helicobacter* spp.	*Actinomyces* spp., CPV, *Mycoplasma* sp., *Chlamydia*, *C. perfringens alpha*, *Helicobacter* spp.
24	Otters 159	*Actinomyces* spp., *Chlamydia*, *C. perfringens alpha*	*Actinomyces* spp., *Chlamydia*, *C. perfringens alpha*
25	Otters 160	*Actinomyces* spp., *Chlamydia* sp.	*Actinomyces* spp., *Chlamydia* sp.
26	Otters 161	*Actinomyces* spp., *Chlamydia*, *Parvovirus*, *C. perfringens alpha*	*Actinomyces* spp., *Chlamydia*, *Parvovirus*, *C. perfringens alpha*
27	Otters 162	*Actinomyces* spp., *Chlamydia* sp., CPV	*Actinomyces* spp., *Chlamydia* sp., CPV
28	Otters 163	*Actinomyces* spp.	*Actinomyces* spp.
29	Otters 164	*Actinomyces* spp.	*Actinomyces* spp.
30	Otters 165	*Actinomyces* spp.	*Actinomyces* spp.

## Data Availability

The datasets used and analyzed in the current study are available from the corresponding authors upon reasonable request.

## Supplementary Materials

The following supporting information can be downloaded at: https://www.mdpi.com/article/10.3390/pathogens15010023/s1, Supplementary File S1: list of pathogens included in the tar-geted NGS pathogen panel.
