# Supplementary material for: MinION Adapted tNGS Panel for Carnivore Pathogens Including SARS-CoV-2
Source: Pathogens. 2025 Dec 24;15(1):23. doi: 10.3390/pathogens15010023 (PMC12844967; doi:10.3390/pathogens15010023)
Supplement: Supplementary file 1 [file pathogens-15-00023-s001.zip › pathogens-4028211-supplementary.pdf]

Supplementary File S1- list of pathogens included in the targeted NGS pathogen panel:

Clostridium perfringens- NetB, NetE, NetF, alpha, beta, beta2, epsilon, iap (iota), cpe

C. difficile- toxin A and B genes

Anaplasma phagocytophilum

Anaplasma platys

EEE

WNV

Rabies- skunk, fox, raccoon

Yersinia pestis

Yersinia enterocolitica

Tularemia

Bartonella spp.

Bartonella henselae

Bartonella quintana

Bartonella vinsonii

Bartonella koehlerae

Bartonella clarridgeiae

Bartonella washoensis

Bartonella rochalimae

Mycoplasma haemocanis

C. Mycoplasma haemominutum

Mycoplasma haemofelis

C. Mycoplasma turicensis

C. Mycoplasma haematoparvum

Brucella spp.

Brucella canis

Ehrlichia canis

Ehrlichia chaffensis

Ehrlichia ewingii

Ehrlichia muris

RMSF

Rickettsia spp.

Rickettsia felis

Rickettsia asembonensis

FeLV

FIV

Salmonella spp.

Lawsonia intracellularis

Tritrichomonas

SARS-CoV-2

Klebsiella pneumoniae

Klebsiella spp.

Axylosoxida

Toxocara canis

Toxocara cati

Streptococcus group D

Hepatozoon felis

Hepatozoon canis

Hepatozoon americanum

Cryptosporidium

Canine parvovirus 2- with typing

Canine bocaparvovirus 1 (minute virus of canines)

Campylobacter jejuni

Giardia

Rotavirus A- canine

Histoplasma capsulatum

Blastomyces dermatitidis

Borrelia burgdorferi

Borrelia garinii

Borrelia hermsii

Borrelia parkeri

Borrelia turicacate

Cytauxzoon felis

E. coli- cnf1, cnf2, sta, eae, stx1, stx2, F5, F41, LT, hlyA, hlyD, papA, ipaH, afaD, sfaS, iutA, bfp

Bornavirus

Enterobacter cloacae

Enterobacter faecalis

Enterobacter faecium

Coccidioides

Staphylococcus aureus

Staphylococcus pseudintermedius

Staphylococcus mecA

Aspergillus spp.

Actinomyces spp.

Serratia spp.

Nocardia spp.

Mycobacterium spp.

Mycobacterium avium complex

Mycobacterium leprae

Pythium

Prototheca spp.

Zygomycetes

Leptospira spp. (pathogenic)

Chlamydia spp.

Chlamydia felis

Chlamydia psittasi

Neospora canis

Neospora hughesi

Mycoplasma spp.

Mycoplasma cynos

Mycoplasma canis

Mycoplasma felis

Listeria monocytogenes

Canine respiratory coronavirus

Canine enteric coronavirus

Canine herpesvirus

Canine distemper virus genotyping based on amplicon coverage of M-F region

Ureaplasma spp.

Bordetella bronchiseptica

Pneumovirus

Canine parainfluenza virus

Canine influenza- H3, N8, N2

Feline calicivirus

Feline herpesvirus

Feline coronavirus (FIP)

Helicobacter spp.

Helicobacter felis

Sporothrix

Canine circovirus

Adenovirus 2

Adenovirus 1

Campylobacter coli

Babesia spp.

Babesia vulpes

Babesia canis canis

Babesia canis vogeli

Babesia canis rossi

Babesia gibsoni

Streptococcus canis

Streptococcus equi zooepidemicus

Streptococcus spp.

Rhodococcus VapA

Angiostrongylus vasorum

Pseudorabies

Leishmania

Cryptococcus

Tetanus

Citrobacter fruendii

Citrobacter koseri

Erysipelothrix rhusiopathiae

Pasteurella canis

Pasteurella multocida

Candida albicans

Candida glabrata

Candida auris

Candida tropicalis

Candida parapsilosis

Bluetongue virus

Streptococcus equisimilis

Rustrela virus

Aleutian mink disease

Mink enteritis

Mink coronavirus

Ferret coronavirus

Feline panleukopenia virus

Skunk amdoparvovirus

Baylisascaris procyonis

Dirofilaria immitis

Trypanosoma cruzi

Feline astrovirus (Mamastrovirus 2)
